# Supplementary material for: Establishment of CRISPR/Cas9-based knock-in in a hemimetabolous insect: targeted gene tagging in the cricket Gryllus bimaculatus
Source: Development. 2025 Jan 7;152(1):dev199746. doi: 10.1242/dev.199746 (PMC11829760; doi:10.1242/dev.199746)
Supplement: Supplementary information [file develop-152-199746-s1.pdf]

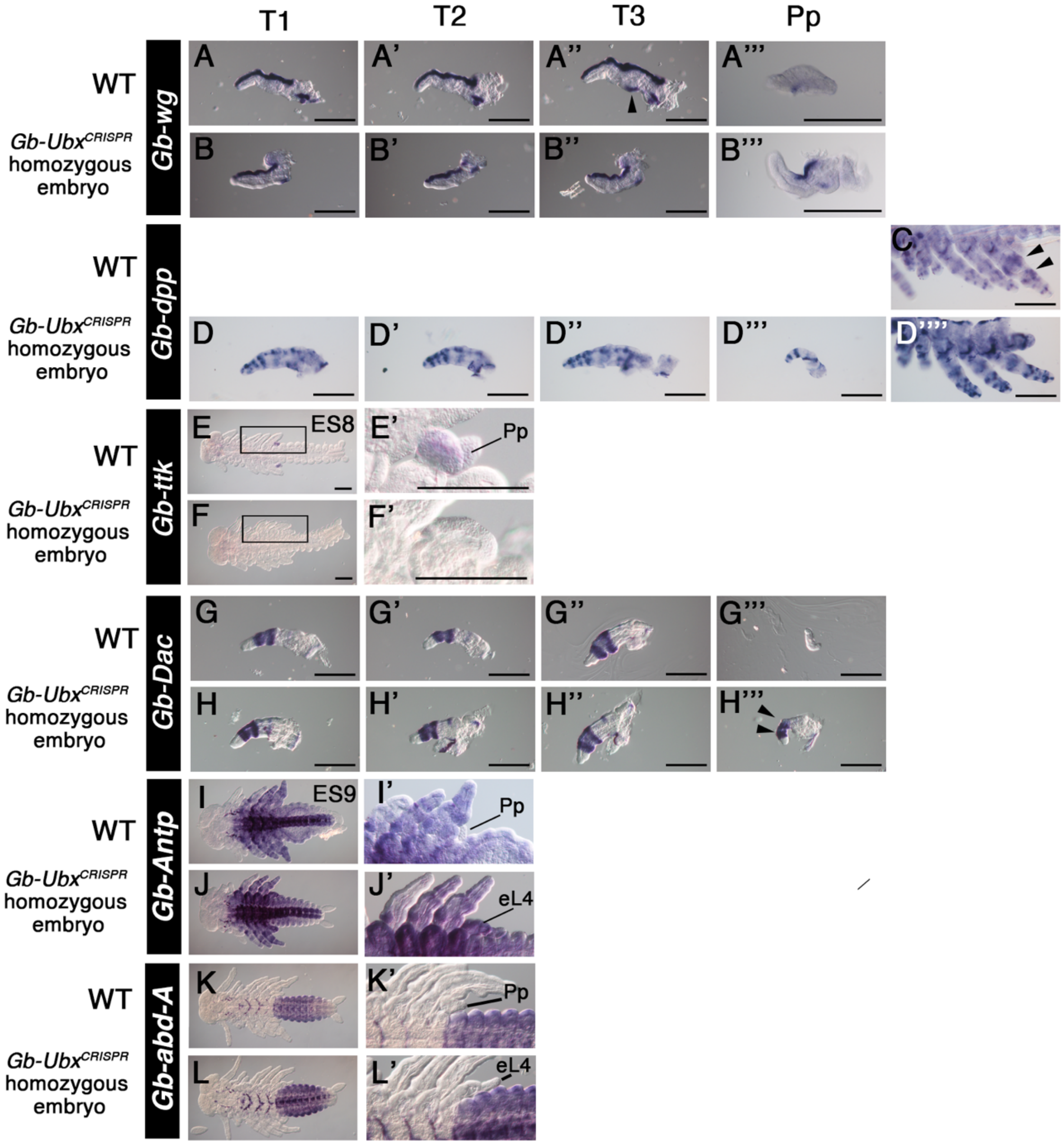

Development • Supplementary information

Fig. S1. Expression pattern of limb patterning genes in *Gb-Ubx*^{CRISPR} homozygous mutants.
To understand the genetic basis of the T3 phenotypes in the *Gb-Ubx*^{CRISPR} homozygous mutants, we examined the embryonic expression pattern of the leg patterning genes *wingless* (*Gb-wg*), *decapentaplegic* (*Gb-dpp*), and *dachshund* (*Gb-dac*). Wild type T3 legs express *Gb-Ubx* (Barnett et al., 2019; Matsuoka et al., 2015; Zhang et al., 2005) and have T3 leg-specific expression of some leg-patterning genes. Namely, *Gb-wg* is expressed on the ventral side of each leg segment (Niwa et al., 2000) and on the dorsal side of the femur in the T3 leg (Supplementary Fig. 1A''), but not in the T1 or T2 legs (Supplementary Fig. 1A-A'). In *Gb-Ubx*^{CRISPR} mutants, dorsal T3 femoral expression of *Gb-wg* was undetectable (Supplementary Fig. 1B''), consistent with a transformation of this appendage towards an anterior fate. *Gb-dpp* is expressed in several dorsal and ventral spots in wild type developing limbs, and in the T3 leg, *Gb-dpp* is expressed in five circumferential bands (Supplementary Fig. 1C; Niwa et al., 2000). In contrast, in *Gb-Ubx*^{CRISPR} embryos, *Gb-dpp* expression in the T3 leg resembled the expression pattern observed in wild type T1 or T2 legs (Supplementary Fig. 1D'' and D'''' compared with Supplementary Fig. 1C). Taken together, the T3 leg-specific pattern of multiple leg-patterning genes was absent, consistent with disruption of the *Gb-Ubx* locus. These results suggest that the T3 segment in *Gb-Ubx*^{CRISPR} mutants acquired characteristics of the T2 segment, consistent with a homeotic transformation loss-of-function phenotype of *Gb-Ubx*^{CRISPR}. The appendage on the A1 segment, called the pleuropodium, is a transient appendage structure only observed during mid-embryogenesis (Rathke, 1844; Wheeler, 1892), and is thought to be involved in secreting hatching enzymes (Konopová et al., 2020; Slifer, 1937). In wild type embryos, *Gb-Ubx* is strongly expressed in this organ at early embryonic stages (Barnett et al., 2019; Matsuoka et al., 2015; Zhang et al., 2005), as are *Gb-Dll* and *tramtrack* (*Gb-ttk*) (Fig. 2E and Supplementary Fig. 1E and E'; Barnett et al., 2019). In *Gb-Ubx*^{CRISPR} mutants, the pleuropodia appeared as small, twisted leg-like structures (Fig. 2E, Supplementary Fig. 1B''', D''', and H''') that lacked *Gb-ttk* expression (Supplementary Fig. 1F and F') and ectopically expressed *Gb-wg*, *Gb-dpp, Gb-Dll*, and *Gb-Dac* in patterns like those observed in wild type thoracic legs (Fig. 2E, Supplementary Fig. 1B''', D''', and H''').We speculate that knock-out of *Gb-Ubx* may cause misexpression of other Hox genes and contribute to the *Gb-Ubx*^{CRISPR} phenotype. To test this hypothesis, we examined the expression patterns of *Antennapedia* (*Antp*) and *abdominal-A* (*abd-A*) in *Gb-Ubx*^{CRISPR} embryos. In wild type embryos *Gb-Antp* is not expressed in the pleuropodia (Supplementary Fig. 1I and I'), while *Gb-abd-A* is expressed in the posterior half of the A1 to A9 segments but not in the pleuropodia (Supplementary Fig. 1K and K'). In *Gb-Ubx*^{CRISPR} embryos, the expression pattern of *Gb-abd-A* was unchanged (Supplementary Fig. 1L and L'), but *Gb-Antp* was misexpressed in the pleuropodia (Supplementary Fig. 1J and J'). We interpret these expression patterns as evidence that *Gb-Ubx* represses *Gb-Antp* expression in the pleuropodia, and the interruption of the *Gb-Ubx* locus causes pleuropodia to be transformed towards thoracic leg identity, which may be induced by misexpression of *Gb-Antp.* Scale bar: 200 µm. Embryonic staging as per (Donoughe and Extavour, 2016).

Development • Supplementary information

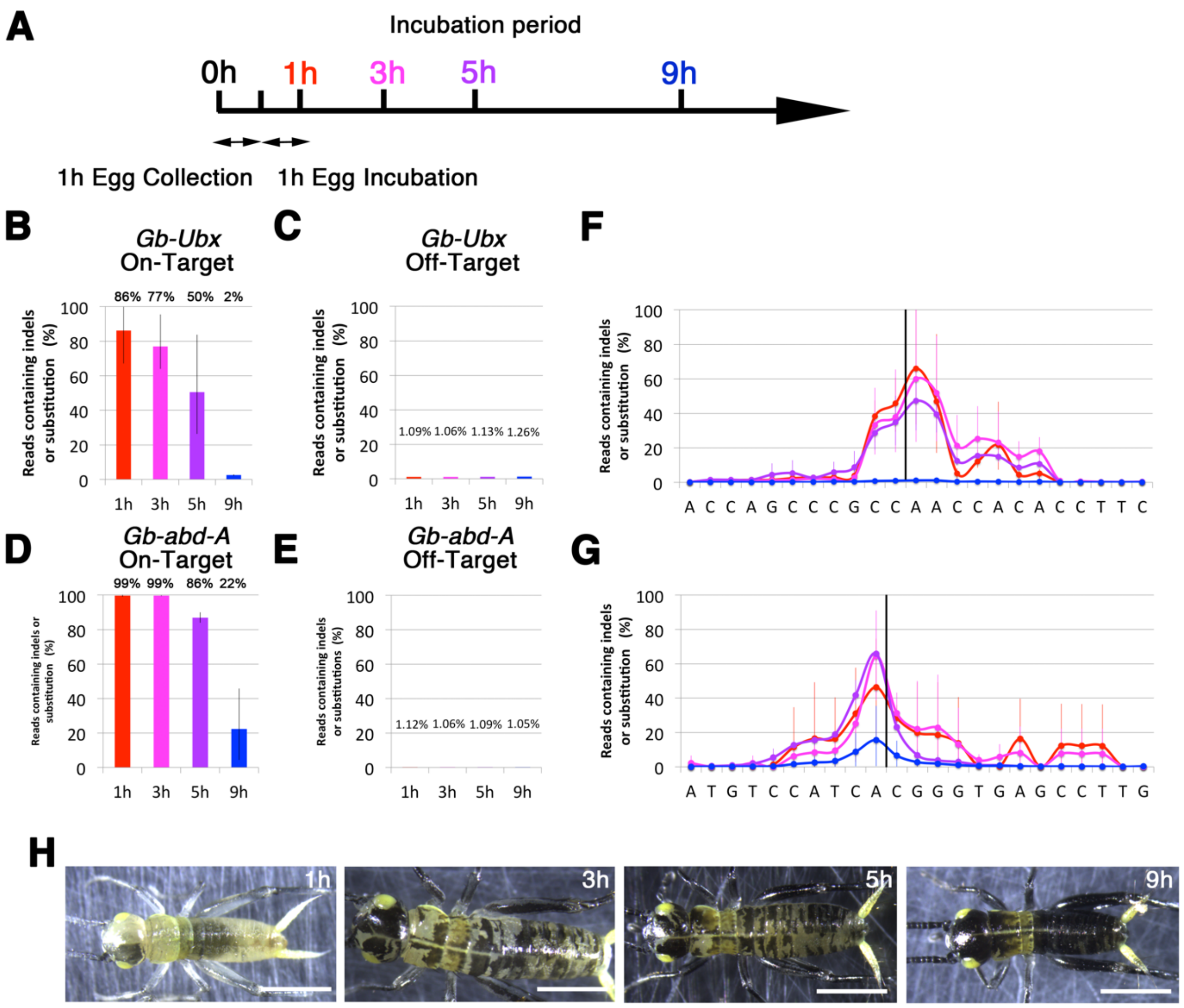

Fig. S2. In-depth analysis of the feature of mutagenesis by CRISPR/Cas9 system in the crickets.

(A) Schematic of injection timeline. After a 1h egg collection and 1h subsequent incubation, eggs were further incubated for 1h, 3h, 5h and 9h, respectively before injection. (B, C) NHEJ mutation rate (indels) at both on-target and off-target sites at the *Gb-Ubx* locus. The NHEJ mutation rate decreased with increasing age of injection and was <1.3% at the studied off-target site. (D, E) NHEJ mutation rate (indels) at both on-target and off-target sites at the *Gb-abd-A* locus NHEJ mutation rate decreased with increasing age of injection and was <1.2% at the studied off-target site. (F, G) Pattern of NHEJ mutations at each injection time point in the sequence around the CRISPR target site. Black bar indicates the site targeted for CRISPR/Cas9-induced double-stranded breaks. The pattern of NHEJ mutations was similar for all injection ages, but the rate decreased with increasing age of injection. (H-H''') Cuticular phenotype of mosaic G_0 hatchlings resulting from injection with *Gb-Lac2* sgRNA (Fig. 1) at each tested injection time point.

Development • Supplementary information

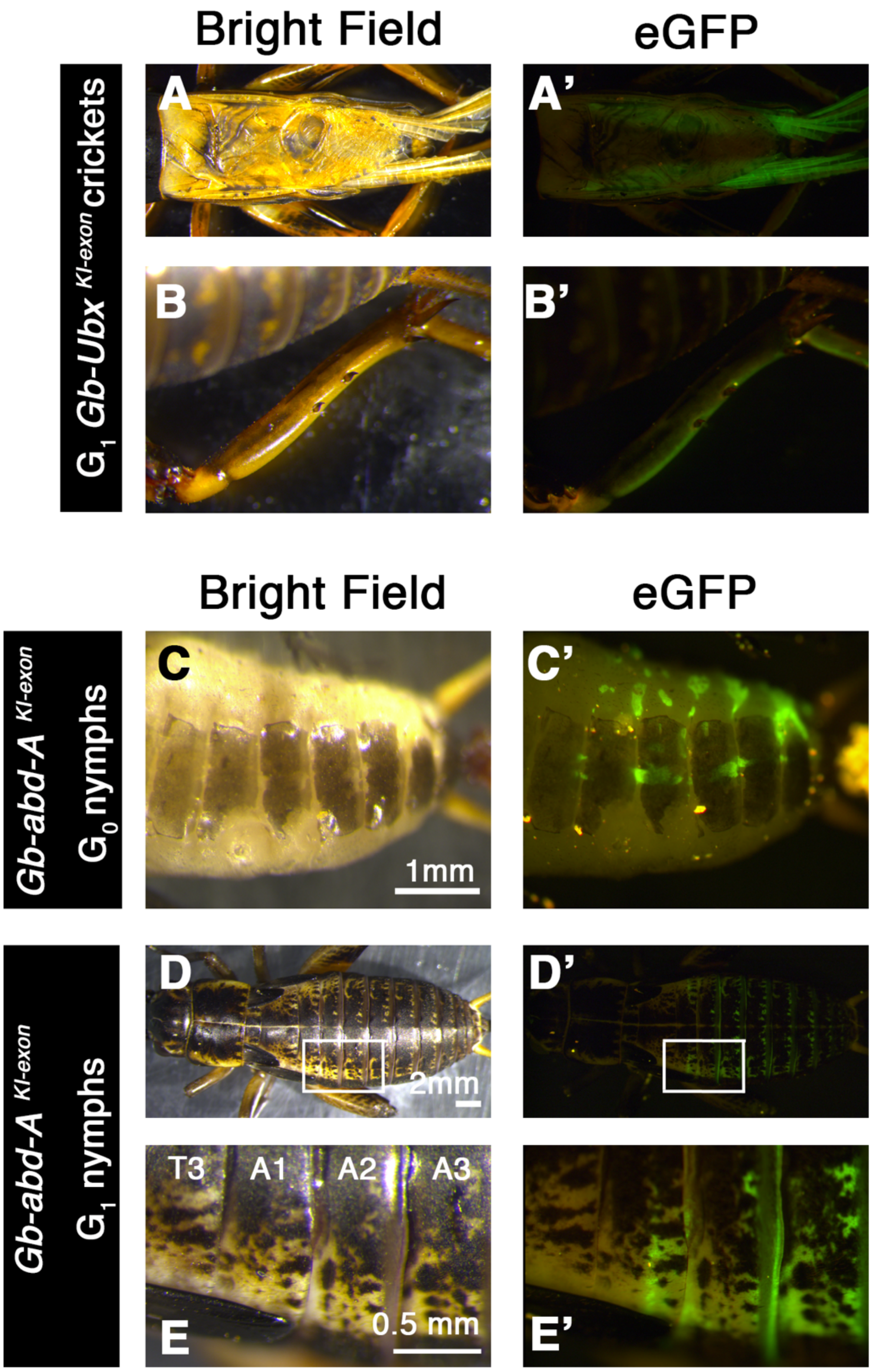

Fig. S3. eGFP expression in the KI crickets.

(A, A') eGFP expression in G_1 *Gb-Ubx*$^{KI\text{-}exon}$ heterozygous male nymphs was visible in the hindwings, which are on the T3 segment. (B, B') *Gb-Ubx*$^{KI\text{-}exon}$ heterozygous adults also showed eGFP expression in T3 legs. (C,C') In G_0 mosaic *Gb-abd-A*$^{KI\text{-}exon}$ nymphs, eGFP expression was detected in patchy cells of the abdominal cuticle. (D, D') In G_1 *Gb-abd-A*$^{KI\text{-}exon}$ nymphs, eGFP expression was observed in the cuticle of A1 to A8, which is similar to the previously reported embryonic expression pattern of *Gb-abd-A* (Barnett et al., 2019; Matsuoka et al., 2015; Zhang et al., 2005). (E,E') Higher magnification of region boxed in white in (D, D').

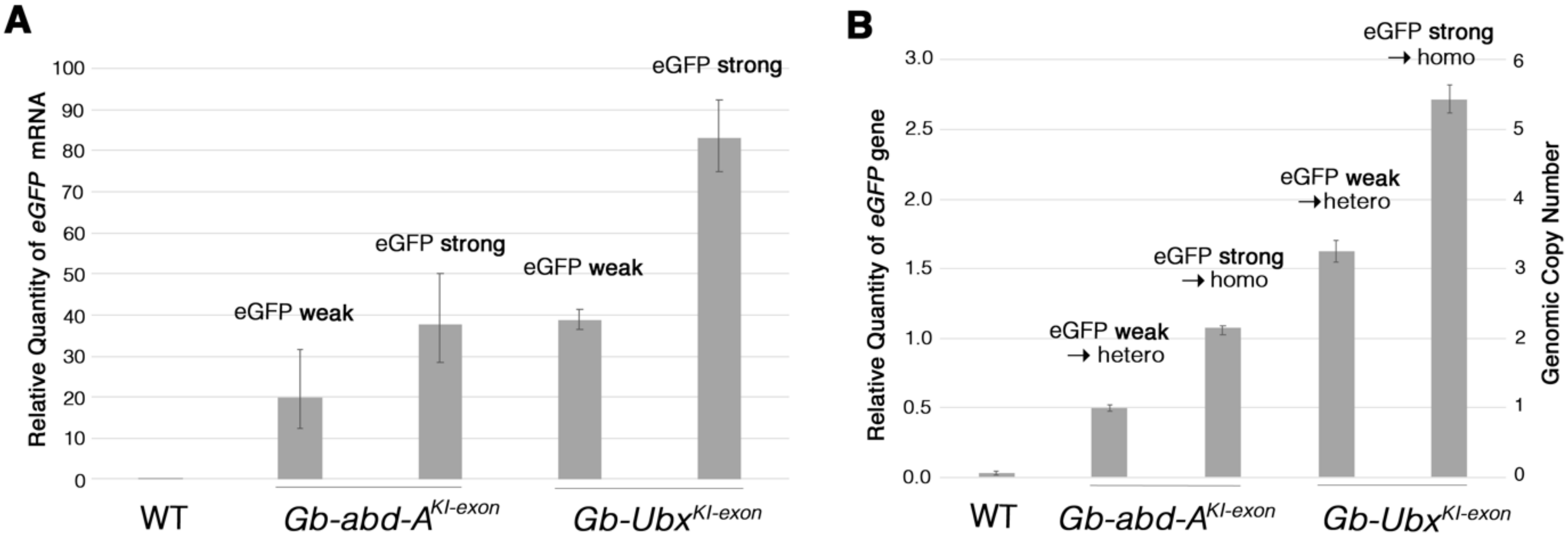

Fig. S4. Expression levels and genomic copy number of the integrated eGFP genes in the KI crickets.

(A) Relative quantity of *eGFP* mRNA in embryos of *Gb-abd-A*$^{KI\text{–}exon}$ and *Gb-Ubx*$^{KI\text{-}exon}$ lines. Embryos of each KI line were divided into two sample groups, respectively, according to the intensity of eGFP fluorescence (eGFP weak/strong). *Gb-beta-actin* was used as an internal standard. n = 5 for each sample group. (B) Estimation of genomic copy numbers of the transgene *eGFP* in the KI individuals by quantitative RT-PCR. The single copy gene *Gb-otd* was used as the reference. The results may indicate that

the *Gb-abd-A*$^{KI\text{–}exon}$ line has one copy of the integrated plasmid, and that the *Gb-Ubx*$^{KI\text{–}exon}$ line has three copies of the integrated plasmid. eGFP weak- and strong-expressing embryos in (A) corresponded to heterozygous and homozygous genotypes for the *eGFP* insertion, respectively.

Development • Supplementary information

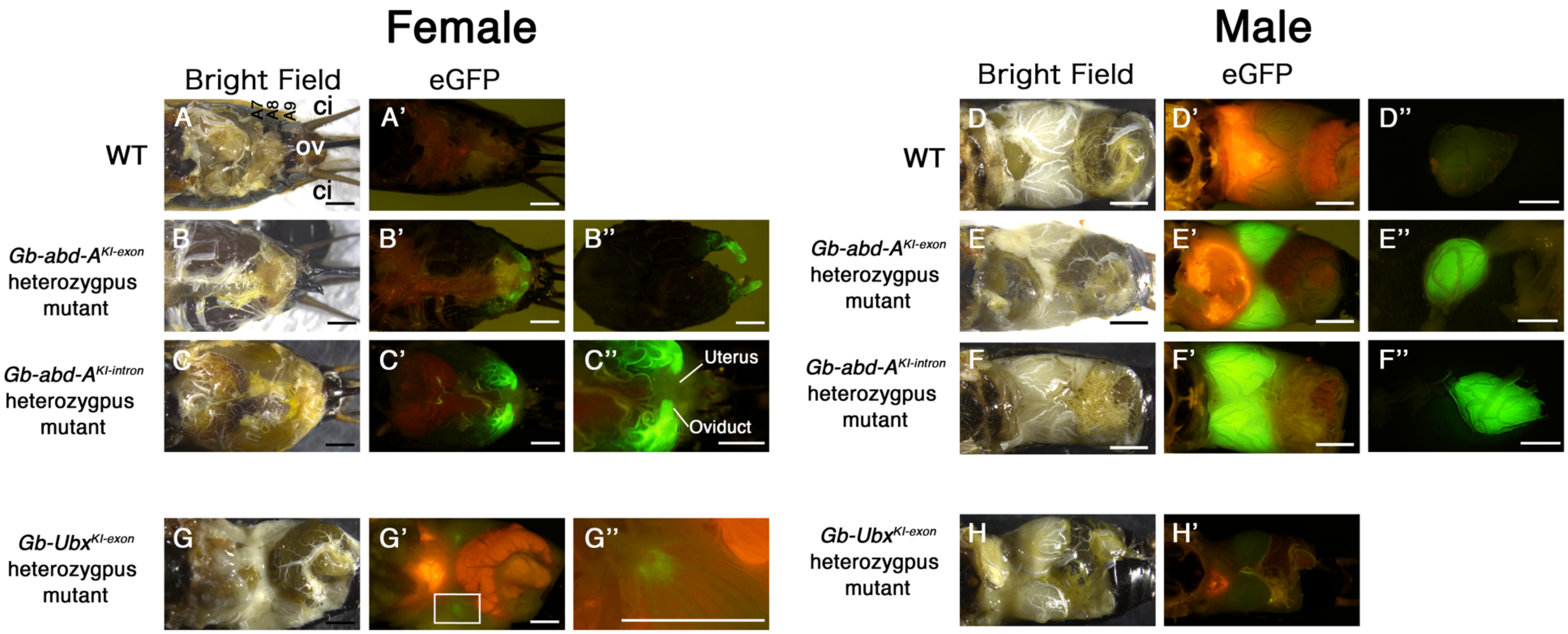

Fig. S5. eGFP expression in genitalia of KI crickets.

(A-C) Adult female viscera in ventral view made visible by removing the cuticle. (A, A') Ventral posterior view of WT adult female abdomen, indicating cerci (ci) and single ovipositor (ov). (B-B'') Dissection revealed that eGFP expression was observed at the posterior tips of the oviducts, and the oviducts were not fused with the uterus (B''). (C, C') In *Gb-abd-A$^{KI\text{-}intron}$* females, eGFP expression in the oviducts was brighter and broader than that detected in *Gb-abd-A$^{KI\text{-}exon}$* females (B'), but the oviducts were fused with the uterus (C''). (D-F) Adult male viscera in ventral view made visible by removing the cuticle. (E-F) Single testes dissected out of the abdominal cavity. In *Gb-abd-A$^{KI\text{-}exon}$* and *Gb-abd-A$^{KI\text{-}intron}$* G_2 adult males, ubiquitous eGFP expression was observed in the testis (E'and F'). (G-G'') *Gb-Ubx$^{KI\text{-}exon}$* females showed eGFP expression at the anterior tip of the ovaries. (H and H') In *Gb-Ubx$^{KI\text{-}exon}$* males, no eGFP expression was detected in the testis. Scale bar: 2 mm.

Development: doi:10.1242/dev.199746: Supplementary information

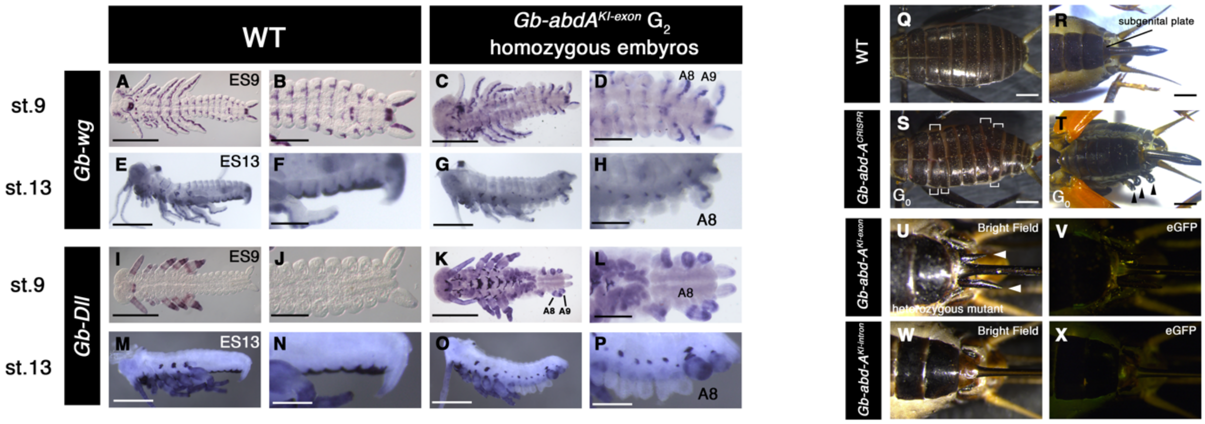

Fig. S6. Developmental and morphological phenotypes in *Gb-abd-A*$^{KI\text{-}exon}$ mutants.

We detected several different phenotypes in *Gb-abd-A*$^{KI\text{-}exon}$ G_0 and G_1 mutants: (1) supernumerary leg-like structures on the abdomen; (2) fusion of cuticle segments on the abdomen; (3) additional ovipositors in heterozygous females. Supernumerary leg-like structures were observed on the abdomen of mosaic G_0 embryos and nymphs. To elucidate the genetic mechanism underlying the production of these structures, we examined the expression patterns of leg patterning genes in homozygous *Gb-abd-A*$^{KI\text{-}exon}$ embryos. (A, B) Expression pattern of *Gb-wg* in ES9 wild type embryos. *Gb-wg* was ectopically expressed along the ventral side of the supernumerary leg-like structures, as in wild type developing limbs, generated on the A8 and A9 (C and D). (E, F) Expression pattern of *Gb-wg* in ES13 wild type embryos. The ectopic *Gb-wg* expression pattern was retained at later stages (G and H). (I, J) Expression pattern of *Gb-Dll* in ES9 wild type embryos. *Gb-Dll* was ectopically expressed in the supernumerary leg-like structures in the A2-A9 segments (K and L), which is similar to the expression pattern observed in the thoracic legs (I). (M, N) Expression pattern of *Gb-Dll* in ES13 wild type G_2 embryos. As the *Gb-abd-A*$^{KI\text{-}exon}$ embryo developed, *Gb-Dll* expression was no longer detected in the supernumerary leg-like structures in the A2 throughA7 segments, but it remained detectable in the supernumerary leg-like structures in the A8 and A9 segments (O and P). Taken together, misexpression of *Gb-Dll* led to the generation of leg-like structures on the early embryonic abdomen, while at later stages, the only structures expressing *Gb-wg* retained the expression of *Gb-Dll*. The strongly affected embryos did not hatch, suggesting that the low hatching rate

Development • Supplementary information

observed in these G0 embryos (Table 3) might be due to this phenotype. Q, R) Wild type nymph abdomen. (S) Approximately 10% of G_0 *Gb-abd-A*$^{KI\text{-}exon}$ nymphs showed fusion of abdominal cuticle in some segments (white brackets). (T) Approximately 10% of G_0 *Gb-abd-A*$^{KI\text{-}exon}$ nymphs showed leg-like structures on the abdomen. (U) G_1 heterozygous *Gb-abd-A*$^{KI\text{-}exon}$ female nymphs showed ectopic ovipositors (arrowheads). In *D. melanogaster iab-4* mutants, one of the *cis*-regulatory regions of *abd-A* affects gonadal development in females (Cumberledge et al., 1992). In severe cases, these mutants failed to form a gonad, and in mild cases, the gonad was formed but the ovary and oviduct junctions were not attached, and thus egg transfer from ovary to the uterus was blocked (Cumberledge et al., 1992). Taken together, these results suggest that the function of *Gb-abd-A* in the development of the adult ovary, specifically in ensuring appropriate joining of the uterus to the oviducts to allow egg release, is conserved between *D. melanogaster* and *G. bimaculatus*. (V) eGFP expression in G_1 heterozygous *Gb-abd-A*$^{KI\text{-}exon}$ nymphs. (W) *Gb-abd-A*$^{KI\text{-}intron}$ nymphs never developed ectopic ovipositors. (X) eGFP expression in G_1 *Gb-abd-A*$^{KI\text{-}intron}$ nymphs. Scale bars: 500 µm in (A) through (P); 2 mm in (Q) through (T). Embryonic staging as per (Donoughe and Extavour, 2016).

Development • Supplementary information

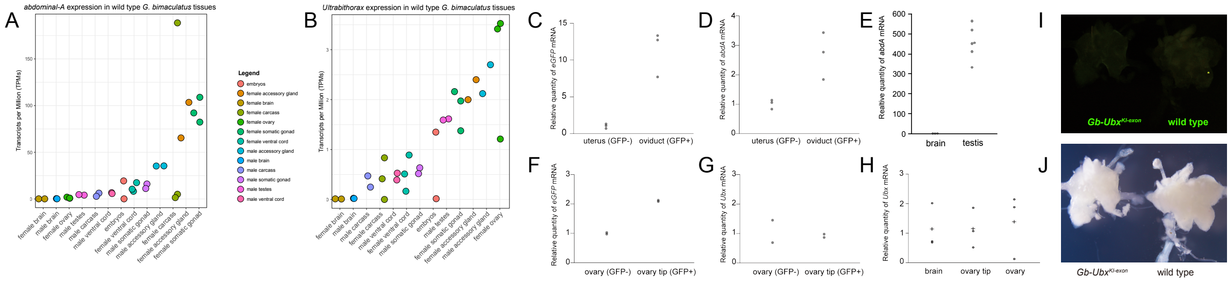

Fig. S7. Relative quantification of *eGFP* expression level in the KI lines. Transcript abundance of *Gb-abd-A* (A) and *Gb-Ubx* (B) in wild type *G. bimaculatus* tissues.

Transcripts per million for each gene per tissue type (X axis categories; colors for each tissue type shown in legend) are shown, derived from previously reported transcripts (Whittle et al. 2021). (C) Relative quantity of *eGFP* in the uterus (eGFP-) and oviduct (eGFP+) from *Gb-abd-A*$^{KI\text{-}exon}$ G_2 adult female. (D) Relative quantity of *Gb-abd-A* in the uterus (eGFP-) and oviduct (eGFP+) from *Gb-abd-A*$^{KI\text{-}exon}$ G_2 adult female. (E) Relative quantity of *Gb-abd-A* in the brain (eGFP-) and testis (eGFP+) from *Gb-abd-A*$^{KI\text{-}exon}$ G_2 adult male. (F) Relative quantity of *eGFP* in the ovary (eGFP-) and ovary tip (eGFP+) from *Gb-Ubx*$^{KI\text{-}exon}$ G_2 adult female. (G) Relative quantity of *Gb-Ubx* in the ovary (eGFP-) and ovary tip (eGFP+) from *Gb-Ubx*$^{KI\text{-}exon}$ G_2 adult female. (H) Relative quantity of *Gb-Ubx* in the brain, ovary (eGFP-) and ovary tip (eGFP+) from *Gb-Ubx*$^{KI\text{-}exon}$ G_2 adult male. (I) Picture of the brain from wild type adult male and *Gb-Ubx*$^{KI\text{-}exon}$ G_2 adult male imaged with blue light fluorescence filter suitable f or revealing eGFP expression. (J) Bright field image of the same brains shown in (I).

Development • Supplementary information

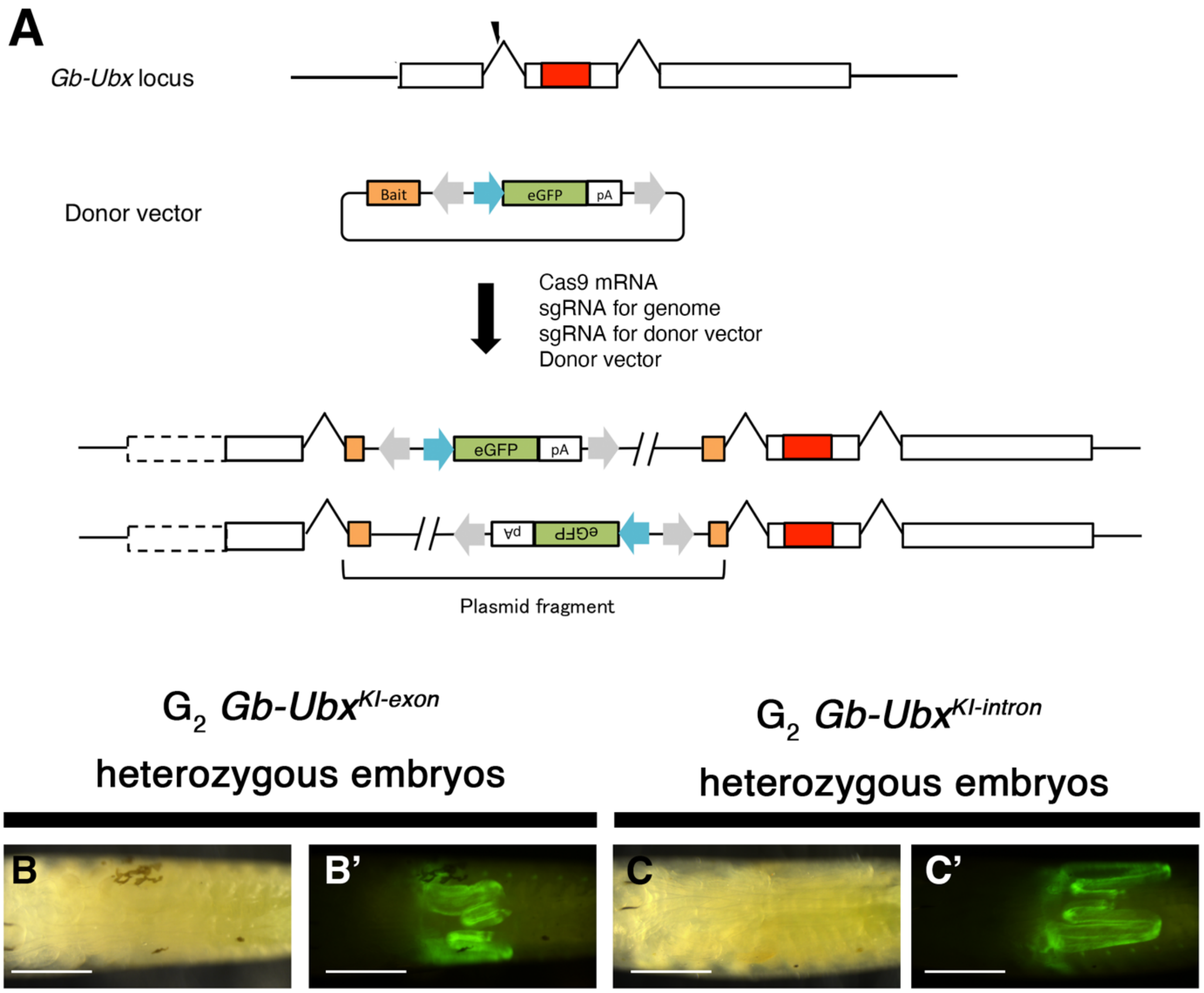

Fig. S8. Knock-in against *Gb-Ubx* intronic region.

(A) Scheme of knock-in experiment targeted to a *Gb-Ubx* intron. White boxes: exons; red box: homeodomain; black arrowhead: sgRNA target site. We used the same donor vector construct as that used in the experiment against *Gb-Ubx* (Fig. 3), substituting a *Gb-Ubx* intron-specific sgRNA. Two patterns of insertion are predicted to occur due to NHEJ. (B, B') Expression pattern of eGFP in G_2 *Gb-Ubx*$^{KI\text{-}exon}$ stage 17 embryos. (C, C') Expression pattern of eGFP in G_2 *Gb-Ubx*$^{KI\text{-}intron}$ stage 17 embryos. *Gb-Ubx*$^{KI\text{-}exon}$ embryos (B') showed shorter legs than G_2 *Gb-Ubx*$^{KI\text{-}intron}$ embryos (C'). Scale bars: 500 µm. Embryonic staging as per (Donoughe and Extavour, 2016).

Development • Supplementary information

Table S1. sgRNA sequences used in this study

sgRNA name	***sgRNA sequence (5'->3')*** (**Bold underline** indicates PAM sequence)
Gb-lac2 exon	GGGGTCCTGGCCCGGGTTGA**CGG**
Gb-Ubx exon	GGGTAGAAGGTGTGGTTGGC**GGG**
Gb-Ubx intron	GGACTGGCCACGCTCCAAGG**AGG**
Gb-abd-A exon	GGGGCAAGGCTCACCCGTGA**TGG**
Gb-abd-A intron	GCTCGCGGTGTTTTACGGCT**GGG**

Table S2. Primers used in this study

Primer name	***Primer seq (5'->3')***
Gb-lac2 genotype Fw	GCGCGACCCCAACAGCAACC
Gb-lac2 genotype Rv	TGGCCCTTGCCGTTGATGAGC
Gb-Ubx exon genotype KO Fw	CGTTTGTGAAACGTATGGCCCGTTA
Gb-Ubx exon genotype KO Rv	GTCCCTGGGCTCCTGGAACACG
Gb-Ubx exon genotype KI genome Fw	AACACGTGCTCCCTCAACTC
Gb-Ubx exon genotype KI genome Rv	TGAAACGTATGGCCCGTTAT
Gb-Ubx exon genotype KI vector 5' Rv	GTCGCATGCTCCTCTAGACTCG
Gb-Ubx intron genotype Fw	GCAGAACCGTTTCATGAATGT
Gb-Ubx intron genotype Rv	ATTCTCGCCCTTATGCAGAG
Gb-abd-A exon genotype Fw	CCGATTCCATGGTGAACTA
Gb-abd-A exon genotype Rv	AGAACGGAACGCAGTGAGTTAG
Gb-abd-A exon genotype vector 3'	GAACTTCAGGGTCAGCTTGC
Gb-abd-A exon genotype vector 5'	CACAAGGCACAAATGCTCGT
Gb-abd-A intron Fw	CGGATCTATTCGGCCATTT
Gb-abd-A intron Rv	TCAAACGGATCTTCCTCTCG
Gb-otd qPCR Fw	CATTCACGTCTCCGCCATAC
Gb-otd qPCR Rv	GCTCCATCAACAGGCAAACA
eGFP qPCR Fw	CAGAAGAACGGCATCAAGGT
eGFP qPCR Rv	GGGTGCTCAGGTAGTGGTTG
Gb-beta-actin qPCR Fw	TTGACAATGGATCCGGAATGT
Gb-beta-actin qPCR Fw	AAAACTGCCCTGGGTGCAT
